# Monocarboxylate transporter 8 deficiency: update on clinical characteristics and treatment

**DOI:** 10.1007/s12020-020-02603-y

**Published:** 2021-03-01

**Authors:** Ferdy S. van Geest, Stefan Groeneweg, W. Edward Visser

**Affiliations:** Academic Center For Thyroid Disease, Department of Internal Medicine, Erasmus Medical Center, Rotterdam, The Netherlands

**Keywords:** MCT8 deficiency, Thyroid hormone signalling, Treatment

## Abstract

Defective thyroid hormone transport due to deficiency in thyroid hormone transporter monocarboxylate transporter 8 (MCT8) results in severe neurodevelopmental delay due to cerebral hypothyroidism and in clinical negative sequelae following a chronic thyrotoxic state in peripheral tissues. The life expectancy of patients with MCT8 deficiency is severely impaired. Increased mortality is associated with lack of head control and being underweight at young age. Treatment options are available to alleviate the thyrotoxic state; particularly, treatment with the thyroid hormone analogue triiodothyroacetic acid seems a promising therapy. This review provides an overview of key clinical features and treatment options available and under development for this rare disorder.

## Characteristics of monocarboxylate transporter 8

Thyroid hormone plays a pivotal role in the regulation of development and metabolic homeostasis of many tissues of the human body. Particularly, adequate thyroid hormone action is crucial for neurodevelopment. Thyroid hormone exerts its cellular action upon binding of triiodothyronine (T3) to its nuclear receptors. The thyroid gland mainly produces the inactive hormone thyroxine (T4); the majority of the bioactive hormone T3 is the product of enzymatic deiodination of T4 [1]. As the active sites of the deiodinating enzymes and the thyroid hormone receptors are expressed intracellularly, both T3 and T4 have to enter the cell by crossing the plasma membrane. This process is mediated by multiple thyroid hormone transporter proteins [2]. Monocarboxylate transporter 8 (MCT8) is among the most efficient and specific thyroid hormone transporters identified to date. MCT8 facilitates cellular uptake and efflux of both T3 and T4, as well as the inactive metabolite reverse T3 [3]. The protein is ubiquitously expressed and has been prominently localized in thyroid, liver, kidneys and the brain [2]. Detailed expression studies have illustrated substantial expression in various region of the developing brain, including glia cells, neurons, the cerebrospinal fluid barriers and, in particular, the blood–brain barrier (BBB) [4]. This latter observation is particularly relevant, as, in the absence of alternative T3 and T4 transporters, MCT8 is the major transporter responsible for transport through the BBB and into neuronal tissues [5, 6].

## Clinical characteristics of MCT8 deficiency

**Fig. 1:**
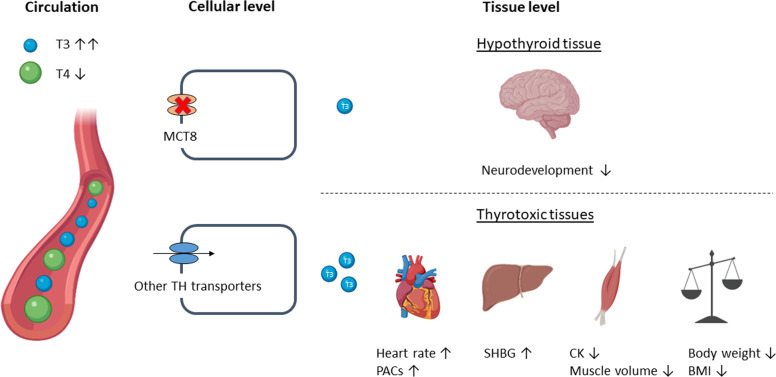
Pathophysiology and clinical characteristics of MCT8 deficiency. Tissues that rely on MCT8 for thyroid hormone transport are relatively hypothyroid, whereas tissues that are independent of MCT8 are in a thyrotoxic state, caused by the increased serum T3 concentrations. TH thyroid hormone, PACs premature atrial complexes, SHBG sex hormone binding globulin, CK creatine kinase, BMI body mass index

Patients harbouring a pathogenic variant in the MCT8 encoding gene *SLC16A2* exhibit severe clinical features known as MCT8 deficiency or Allan–Herndon–Dudley syndrome [7–9]. Different types of mutations underlie this disease (e.g., missense variants, frameshifts or large deletions), resulting in different degrees of impact on transport function, ranging from full to partial deficiency of thyroid hormone transport. Defective MCT8 results in disrupted cerebral thyroid hormone homeostasis, leading to abnormal neurodevelopment. However, the high circulating T3 concentrations cause thyrotoxicosis in MCT8-independent tissues. Thus, the combination of tissue-specific hypothyroid and thyrotoxic features determine the clinical manifestations of this disorder (Fig. 1). For an overview of the main mechanisms of disease, the reader is referred to recent reviews [2, 10, 11]. This review will focus on important clinical characteristics of MCT8 deficiency and provides an overview of treatment options available and under development.

Mild-to-moderate intellectual disability and severe delay in motor development (including truncal hypotonia and lack of head control) are highly prevalent features of the disease [12, 13]. In addition to the neurodevelopmental delay, peripheral hypertonia due to dystonia and spasticity and persistent primitive reflexes are found in the majority of patients. A minority of patients shows a less severe phenotype, exemplified by attaining better motor skills such as head control and the ability to sit independently [13, 14]. Importantly, in general, improvement in development upon aging is limited [9, 12].

**Fig. 2:**
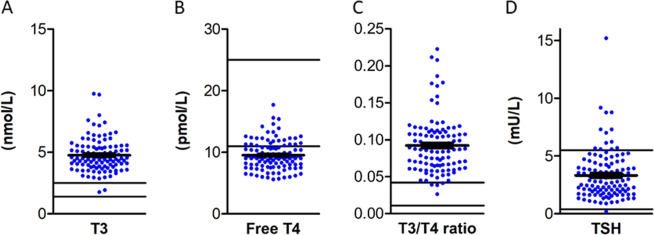
Mean ± SEM (black lines) serum concentrations of T3 (panel (**A**)), free T4 (panel (**B**)), mean ± SEM (black lines) T3/T4 ratio (panel (**C**)) and serum concentrations of thyroid stimulating hormone (TSH) (panel (**D**)). Blue dots represent measurements in individual patients and grey areas the normal range

Serum T3 concentrations are increased above the age-specific reference interval in nearly all patients (Fig. 2A). Serum free T4 concentrations are below the age-specific reference interval in the majority of patients (Fig. 2B). The subsequent high T3/T4 ratio distinguishes MCT8 deficiency from some, but not all other inborn thyroid hormone signalling disorders (Fig. 2C). In particular, patients with resistance to thyroid hormone syndrome alpha show comparable T3/T4 ratios [15, 16]. Serum TSH concentrations are within the age-specific reference interval in most patients (Fig. 2D). Of note, serum T4 concentrations are less decreased in those patients who are less severely affected.

Following the elevated T3 concentrations, clinical and biochemical features resemble a state of chronic thyrotoxicosis. Serum sex hormone binding globulin (SHBG) concentrations, reflecting hepatic thyroid hormone action, are elevated in the majority of patients. Serum creatinine concentrations, reflecting renal thyroid hormone action, and creatine kinase concentrations, reflecting thyroid hormone action in muscles, are often found to be low within the reference range.

Severe underweight is commonly observed in patients with MCT8 deficiency, necessitating the implementation of a feeding tube. Moreover, clinical cardiovascular signs of peripheral thyrotoxicosis, including elevated systolic blood pressure, premature atrial complexes and tachycardia in rest, are highly prevalent in patients with MCT8 deficiency.

In magnetic resonance imaging of patients with MCT8 deficiency, delayed myelination and abnormal cerebral white matter volume are key findings [10, 12, 17].

**Fig. 3:**
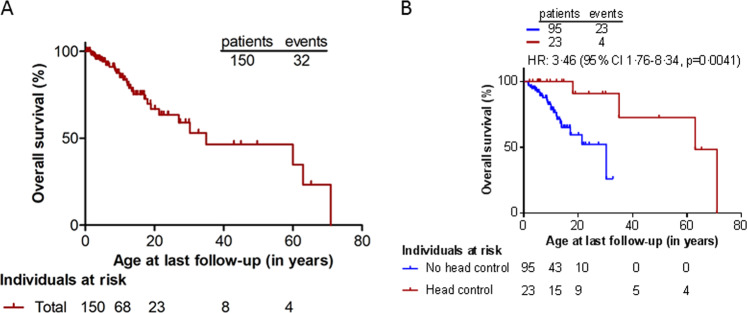
Overall survival of a cohort of 150 patients with MCT8 deficiency (Kaplan–Meier estimates) (**A**). Kaplan–Meier estimates of MCT8-specific survival in patients who attained head control (red line) by the age of 1.5 years versus those who did not (blue line) (**B**)

Recently, it was shown that MCT8 deficiency has a large impact on life expectancy [12]. Median overall survival was strongly reduced (~35 years) (Fig. 3A), with ~30% of patients dying in childhood. A lack of head control at the age of 1.5 years or being underweight at a young age was associated with increased risk for death. The most common causes of death were pulmonary infection and sudden death, together explaining nearly half of the cases. The features of cardiovascular pathology identified in a large proportion of patients might underlie the high number of sudden deaths.

## Clinical management and therapeutic options

**Fig. 4:**
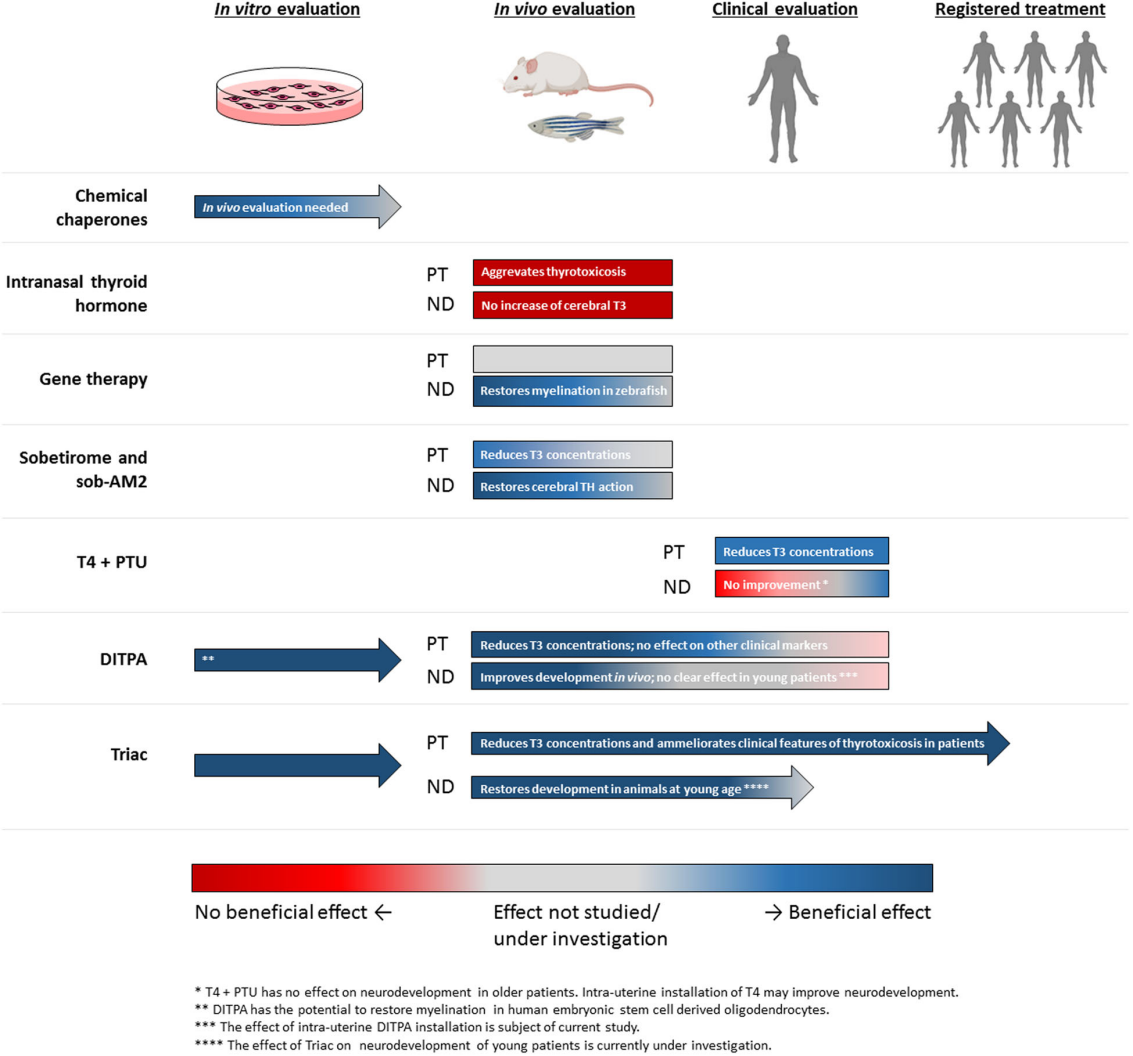
Schematic representation of the development of (potential) therapies for MCT8 deficiency. Arrows indicate that therapies can be/are being evaluated in the next stage. For in vivo and clinical evaluation, treatment effect is divided in effects on peripheral thyrotoxicosis and neurodevelopmental delay. The median age of at clinical evaluation was 1.7 years (range 0.0–38.0) for T4 + PTU, 2.1 years (range 0.7–2.1) for DITPA and 7.1 years (range: 0.8–66.8) for Triac. PT peripheral thyrotoxicosis, ND neurodevelopmental delay

Currently, no registered treatment for MCT8 deficiency is available. However, major advances in the field have taken place over the last decade (Fig. 4). Therapeutic options and (potential) treatment strategies can be divided in three different categories: (1) conventional therapies including supportive care and T4 administration, (2) therapies aiming to recover MCT8 function and (3) treatments aiming to restore thyroid hormone signalling independent of MCT8. As foetal brains in MCT8 deficiency already show disturbed development [18], intervening as early in life as possible seems to be crucial to improve neurodevelopment.

### Conventional therapies

As a first major treatment entity, conventional therapies including supportive care options should be considered. Different supportive strategies can be used to alleviate common features of MCT8 deficiency (e.g., physical therapy to prevent contractures and specialized orthopaedic care for scoliosis). The high prevalence of underweight and muscle wasting and the positive association between being underweight early in life and increased mortality risk stress the importance of optimizing feeding status of these vulnerable patients, e.g., by ways of utilization of a feeding tube, speech therapy to prevent aspiration and professional dietary advice to prevent malnutrition. Together, these interventions might improve the nutritional status of these patients and decrease the risk of aspiration-related death. As cardiovascular abnormalities are common, but often not evaluated in clinical praxis [12], cardiovascular screening might identify preventable or treatable risk factors underlying sudden death. Hence, optimizing nutritional care and other supportive care measures using a multidisciplinary approach is critical in the daily care for patients with MCT8 deficiency.

As peripheral deiodinase activity is increased in MCT8 deficiency, oral administration of T3 and T4 results in aggravation of the peripheral thyrotoxicosis, without improvement of the neurodevelopment [19]. However, when T4 treatment is combined with propylthiouracil (PTU), inhibiting peripheral deiodinase 1 activity, serum T3 concentrations normalize, resulting in decreased thyroid hormone action in peripheral tissues. Moreover, this strategy might theoretically allow T4 to enter the brain via other routes than MCT8-mediated transport. Unfortunately, the effects of this strategy have only been reported for patients who were treated for a short duration or were too old at treatment initiation to expect any improvements in neurodevelopment [2]. Refetoff et al. [20] recently reported about treatment of a foetus with MCT8 deficiency with T4 installations during intra-uterine life and with PTU and T4 combination therapy starting directly after birth. A remarkable improvement in neurodevelopment was observed at 2.5 years of age compared to his older, untreated brother harbouring the same mutation. Thus, intra-uterine installation of therapeutic compounds might be a promising strategy to improve neurodevelopmental outcomes in MCT8 deficiency, although this is limited to a very small group of patients who are diagnosed before birth.

### Therapies aiming to recover MCT8 function

Different animals models of MCT8 deficiency are available (e.g., mouse, zebrafish, chicken) that have helped to understand the mechanisms of MCT8 deficiency as well as to explore therapeutic strategies; it is relevant to realize the advantages and disadvantages of the different mouse models [2]. Mct8 knock-out (KO) mice resemble well the peripheral phenotype, but not the neurodevelopmental phenotype. This is due to redundant expression of the T4 transporter organic anion transporter protein (OATP) 1C1 in the murine BBB. This allows sufficient entry of T4 into the brain for local conversion to T3 and, consequently, Mct8 KO mice lack a gross neuromotor phenotype. In contrast, Mct8/Oatp1c1 double KO (DKO) mice reflect both the peripheral and neurodevelopmental phenotype of MCT8 deficiency and are, therefore, considered the most accurate murine model available [2, 11].

A second important potential treatment entity is to restore MCT8-mediated thyroid hormone transport into the brain. Iwayama et al. investigated whether intravenous (IV) or intracerebroventricular (ICV) injection of human MCT8 (hMCT8) cDNA using adeno-associated virus 9 as a vector could restore cerebral MCT8 expression, improve T3 content and expression of the T3-responsive gene *Hairless* in *Mct8* KO mice. Upon injection via both routes, both cerebral hMCT8 mRNA and protein expression were strongly increased. In contrast to ICV injection, IV injection resulted in an increase of cerebral T3 content. Consequently, cerebral *Hairless* expression improved in IV injected mice, indicating a (partial) restore in thyroid hormone signalling in the brain. Using a similar approach, Zada et al. [21] showed that hypomyelination can be completely recovered by inducing Mct8 expression in the vascular endothelial cells of *mct8*^*−/−*^ zebrafish larvae 10 days post fertilization. Additional studies in mice resembling the neuromotor phenotype and studies focussing on timing, dosing and off-target effects are warranted to explore the potential of this form of gene therapy in human and to determine whether this form of therapy is able to restore thyroid hormone signalling in patients with MCT8 deficiency.

Another potential therapeutic strategy currently under investigation involves the utilization of chemical chaperones. These molecules are capable of altering stability of misfolded proteins and improving trafficking to the cell membrane. Due to the nature of their mode of action, chaperones can selectively target proteins with a reduced surface expression and residual transport capacity. Different chaperones have been tested on in vitro and ex vivo models (e.g., p.F501del as a prototypic mutation), but additional evaluation in appropriate animal models is needed [22–24]. The therapeutic potential of chaperone therapy for patients remains to be explored in selected patients, whose mutations might be responsive to this treatment.

### Therapies aiming to restore thyroid hormone signalling independent of MCT8

The third major group comprises strategies aiming to restore thyroid hormone signalling in an MCT8-independent manner, using different principles. As the intranasal route to the brain does not include the BBB, it was hypothesized that intranasal thyroid hormone administration might restore cerebral thyroid hormone concentrations in MCT8 deficiency. Grijota-Martínez et al. [25] showed that intranasal administration of thyroid hormone is unable to improve cerebral thyroid hormone homeostasis, while plasma T3 concentrations rather further increased.

In contrast, molecules capable of bypassing MCT8 at a cellular level and stimulating the endogenous thyroid hormone receptors, called thyroid hormone analogues, hold great therapeutic potential [26]. Treatment with thyroid hormone analogues has been extensively investigated in different preclinical models mimicking MCT8 deficiency. Moreover, the effects of treatment with thyroid hormone analogues on patients with MCT8 deficiency have been explored in clinical setting.

Preclinical studies in *Mct8/Dio2* DKO mice have shown that thyroid hormone analogue sobetirome and its brain-specific prodrug sob-AM2 are able to (partially) restore cerebral thyroid hormone signalling upon treatment, illustrated by normalization of cerebral *Hairless* expression [27]. Further studies on efficacy and toxicity are needed before this therapy can be evaluated in a clinical setting.

The effects of thyroid hormone analogue 3,5-diiodothyropropionic acid (DITPA) have been evaluated in *Mct8* KO mice and patients with MCT8 deficiency. In these mice, DITPA was shown to enter the brain in an MCT8-independent manner and to correct the thyrotoxic state in peripheral tissues [28, 29]. Moreover, Lee et al. [30] showed that in human embryonic stem cell derived oligodendrocytes DITPA has the potential to restore myelination. In line with the findings in *Mct8* KO mice, in four DITPA-treated patients, serum T3 concentrations normalized upon treatment with DITPA, without aggravation of the hypothyroxinemia [31]. However, the patients did not show improvement in their neurodevelopment or other clinical markers, including body weight.

Triiodothyroacetic acid (Triac) is a thyroid hormone analogue which is produced endogenously by conversion of T3 to its acetic acid derivative. T3-dependent neural differentiation in the cerebral and cerebellar cortex of *Mct8/Oatp1c1* DKO mice improved with a low dose Triac and normalized with a high dose Triac [32, 33]. In addition, Triac completely rescued the number of oligodendrocytes, an indicator of myelination, of *mct8*^*−/−*^ zebrafish larvae 10 days post fertilization. In contrast, DITPA partially rescued the number of oligodendrocytes in these larvae [21]. Following these preclinical studies, an international clinical trial was initiated to determine the effect of Triac on patients with MCT8 deficiency. Forty-six patients (median age 7.1 years, range 0.8–66.8) were treated with Triac for 12 months and a subset of patients continued treatment in a treatment-extension protocol. Serum T3 concentrations were reduced in all patients upon 12 months of treatment [34]. Consequently, improvements in body weight, heart rate and peripheral markers of thyroid hormone signalling (SHBG, creatine kinase, creatinine) were observed in patients who completed the treatment period of 12 months. As severe underweight and cardiovascular anomalies are important clinical features of the peripheral thyrotoxicosis of MCT8 deficiency and underlie substantial morbidity and mortality, amelioration of these thyrotoxic features likely benefits patients with MCT8 deficiency at all ages. Furthermore, a trend of neurodevelopmental improvement was identified in a subset of patients who were treated with Triac early in life.

## Future perspectives

Linked to the European Reference Network on Rare Endocrine Conditions, an international registry for patients with MCT8 deficiency has been started. This initiative aims to identify the needs of patients, parents and physicians and will further inform clinical management and may guide future development of therapies.

Despite major advances in understanding MCT8 deficiency, the efficacy of only one therapy, Triac, has been studied in a clinical trial. Further investigation in preclinical studies is needed to develop other potential therapies. Two research initiatives with different thyroid hormone analogues have recently been initiated. As DITPA is capable of crossing the placenta [35] and may restore brain development [30], mothers who are pregnant of a foetus with MCT8 deficiency can be treated with intra-uterine installation of DITPA on compassionate use basis (NCT04143295). An international phase 2 trial with sites in Europe and the USA investigates the effect of Triac on neurodevelopment when treatment is commenced early in life (NCT02396459). These research initiatives will address the question whether these approaches are able to positively modulate neurodevelopment in patients with MCT8 deficiency.

## Conclusion

Although patients with MCT8 deficiency show an obvious neurodevelopmental phenotype, clinical care should also pay attention to the peripheral thyrotoxic phenotype. Life expectancy is severely decreased compared to healthy individuals and the severity of the peripheral thyrotoxicosis appears to correlate with increased mortality. This is particularly relevant, as Triac treatment, which reduces peripheral thyroid hormone signalling, is available. Therapeutic options to improve neurodevelopment remain limited with a clinical trial with Triac assessing improvement in neurocognitive outcomes underway. Given the complexity and rarity of MCT8 deficiency, it remains pivotal to combine and centralize expertise and efforts of researchers and clinicians over the globe, in order to develop novel effective treatment strategies.
